# Dementia and sexuality in long-term care: Incompatible bedfellows?

**DOI:** 10.1177/14713012211056253

**Published:** 2021-12-14

**Authors:** Alisa Grigorovich, Pia Kontos, Ann Heesters, Lori Schindel Martin, Julia Gray, Laura Tamblyn Watts

**Affiliations:** Department of Recreation and Leisure Studies, 7961 Brock University, St. Catharines, ON, Canada; KITE Research Institute, Toronto Rehabilitation Institute – University Health Network, Toronto, ON, Canada; KITE Research Institute, Toronto Rehabilitation Institute – University Health Network – University Health Network, Toronto, ON, Canada; Dalla Lana School of Public Health, University of Toronto, Toronto, Canada; Bioethics, 7989 University Health Network, Toronto, ON, Canada; Education Investigator 2, TIER (The Institute for Education Research), University Health Network, Toronto, ON, Canada; Assistant Professor, Dalla Lana School of Public Health, University of Toronto, Toronto, ON, Canada; Joint Centre for Bioethics, University of Toronto, ON, Canada; Daphne Cockwell School of Nursing, 104269 Ryerson University, Toronto, ON, Canada; Department of Health & Society, University of Toronto Scarborough, Scarborough, ON, Canada; CanAge, Canada’s National Seniors’ Advocacy Organization, Toronto, ON, Canada

**Keywords:** stigma, intimacy, sexual expression, education, public policy

## Abstract

Despite the recognized benefits of sexual expression and its importance in the lives of people living with dementia, research demonstrates that there are multiple barriers to its positive expression (e.g., expression that is pleasurable and free of coercion, discrimination, and violence) in RLTC homes. These barriers constitute a form of discrimination based on age and ability, and violate the rights of persons living with dementia to dignity, autonomy, and participation in everyday life and society. Drawing on a human rights approach to dementia and sexual expression, we explored the experiences of diverse professionals, family members, and persons living with dementia with explicit attention to the ways in which macro-level dynamics are influencing the support, or lack thereof, for sexual expression at the micro level. Focus groups and in-depth interviews were conducted with 27 participants, and the collected data were analyzed thematically. While all participants acknowledged that intimacy and sexual expression of persons living with dementia should be supported, rarely is such expression supported in practice. Micro-level factors included negative attitudes of professionals toward sexual expression by persons living with dementia, their discomfort with facilitating intimacy and sexual expression in the context of their professional roles, their anxieties regarding potential negative reactions from family members, and concerns about sanctions for failing to prevent abuse. In our analysis, we importantly trace these micro-level factors to macro-level factors. The latter include the cultural stigma associated with dementia, ageism, ableism, and erotophobia, all of which are reproduced in, and reinforced by, professionals’ education, as well as legal and professional standards that exclusively focus on managing and safeguarding residents from abuse. Our analysis demonstrates a complexity that has enormous potential to inform future research that is critically needed for the development of educational initiatives and to promote policy changes in this area.

## Introduction

Sexual expression in later life covers a wide spectrum of activities and experiences related to sexuality, romance, and sexual identity, ranging from physical forms of intimacy to emotional and intellectual closeness (Mahieu & Gastmans, 2015). The most common examples of sexual expression in residential long-term care (RLTC) homes are grooming and self-presentation, hand holding, kissing, and other less physically intense expressions (Frankowski & Clark, 2009; Mahieu & Gastmans, 2015). Sexual expression in later life has been linked with psychological and physiological benefits, including improved quality of life, lower rates of depressive symptoms, and lower risk of certain cancers and fatal coronary events (Smith et al., 2019; Syme, 2014). The emerging, but scant, qualitative research with persons living with dementia in RLTC homes and their spouses builds on this scholarship by highlighting that love, romance, and emotional and physical intimacy, are important aspects of their lives and relationships (Roelofs et al., 2021, 2019; Sandberg et al., 2020).

Despite the benefits of sexual expression and its importance in the lives of people living with dementia, research demonstrates that there are multiple barriers to its positive expression (e.g., pleasurable and free of coercion, discrimination, and violence) in RLTC homes, both at the micro (e.g., negative attitudes and practices of healthcare professionals) and macro (e.g., cultural stigma, professional standards, and public policies that do not recognize importance of positive sexual expression) levels. For example, research on nurses’ and other direct care professionals’ perceptions and responses to the sexual expressions of residents with and without dementia found that they tend to respond to the sexual expressions of persons living with dementia with expressions of disgust, denial, and discouragement (Cornelison & Doll, 2013; Howard et al., 2019; Roach, 2004; Ward et al., 2005). When confronted with overt sexual desires or activity, many professionals report feeling shame and discomfort, as well as the need to intervene in the sexual expression (Roach, 2004; Shuttleworth et al., 2010; Villar et al., 2018). Heteronormative assumptions regarding gender roles and sexual conduct further influence professionals’ perceptions of sexual expression of people living with dementia. For example, the sexual expressions of men are more likely to be eroticized and perceived as dangerous to others, while the sexual expressions of women are more likely to prompt a protective response (Sandberg et al., 2020). There is also evidence that professionals lack cultural competency in sexual and gender diversity and that they may hold more negative perceptions of the sexual expressions of LGBTQ people living with dementia (Caceres et al., 2020). Research with persons living with dementia and their family members has confirmed a lack of support from professionals for sexual expression in the form of negative attitudes and practices, particularly in relation to not respecting sexual privacy and by actively discouraging sexual expression or by avoiding conversation about sexual health and intimacy needs (Roelofs et al., 2021, 2019).

There are some studies that have also identified macro-level barriers to positive sexual expression in RLTC homes. For example, there is a notable absence of attention to sexual expression within educational programs and in the existing standards for professionals, as well as in organizational policies for RLTC homes (Brassolotto et al., 2020; Cornelison & Doll, 2013; Makimoto et al., 2015). Very few RLTC homes have sexual expression policies that protect residents’ rights to intimacy or sexual privacy (Brassolotto et al., 2020; Cornelison & Doll, 2013; Roelofs et al., 2021, 2019; Shuttleworth et al., 2010). Indeed, this explains the notable absence in the literature of any systematic implementation of promising practices with respect to sexual expression. In the absence of clear policy directives and appropriate professional education, professionals tend to rely on their personal values and judgments (Roelofs et al., 2021, 2019; Shuttleworth et al., 2010), which largely reflect and perpetuate cultural stigma (a macro-level barrier) associated with sexual expression outside of boundaries of heteronormative understandings of sexuality (Bauer et al., 2013; Jones, 2020; Simpson et al., 2015; Ward et al., 2005). In the context of dementia, cultural stigma is also produced and reproduced by biomedicalization and “the loss of self discourse” (Herskovitz, 1995; Kontos, 2012), which reinforce the cultural imaginary that persons living with dementia are incapable of purposeful and meaningful communication and the pursuit of life-enhancing relationships and activities. The actions, needs, and movements of persons living with dementia are thus often dismissed or portrayed as aimless in mass media, academic, and policy documents and are frequently perceived as disease-driven “symptoms” or “responsive behaviors” rather than meaningful attempts to communicate or to engage with others (Dupuis et al., 2012; Grigorovich & Kontos, 2018; Kontos et al., 2020).

Stigma associated with aging and disability further extends to the macro-level of legislation that informs the boundaries of legal sexual expression and care of persons living with dementia. For example, many jurisdictions have criminal statutes that define sexual abuse as sex without ongoing verbal consent and/or sex with an individual who is considered to be (or assessed as) incapable of providing consent (e.g., based on their age and/or cognition) (Goldberg, 2018). Moreover, many jurisdictions also have reporting requirements for healthcare professionals that mandate reporting of all cases of suspected and/or actual sexual abuse (Grigorovich & Kontos, 2020; Quinlan & Smele, 2017). While these types of legislation were not explicitly implemented to restrict positive sexual expression of persons living with dementia, they pose a significant barrier to such expression by reinforcing ableism and ageism (Fischel & O'Connell, 2015; Goldberg, 2018; Peisah et al., 2021). In the context of the risk-averse culture of RLTC, such legislation legitimizes the default practice of restricting sexual expression of people with dementia based on the belief that it is unintentional and/or not consensual, and thus always harmful (Howard et al., 2019; Sandberg et al., 2020).

While the existing literature in this area has importantly identified barriers to sexual expression, it falls short of capturing the complexity of this phenomenon in that limited attention has been given to the role of macro factors in influencing micro-level perceptions and practices. Thus, it is our contention that a human rights-based approach is a more productive way forward because it treats the support of positive sexual expression as a matter of social justice that governments have the obligation to attend to using law and public policy. With a human rights approach to dementia and sexuality, the barriers that persons living with dementia experience are treated as a form of discrimination based on age and ability, and attention is focused on the denial of their human rights to dignity, autonomy, and participation in everyday life and society (Grigorovich & Kontos, 2018; Kontos et al., 2016; Peisah et al., 2021). This is because a human rights approach recognizes that all persons, regardless of their abilities and age, have an entitlement to experience positive sexual expression on an equal footing, and it is implied that states and their institutions have an obligation “to act in ways that do not produce or perpetuate barriers to equality and the equal enjoyment of [sexual] rights, including the highest attainable standard” (Miller et al., 2015, p. 22). Such an approach has already been used in dementia studies to argue for the need to support sexual expression for human flourishing as this is related to fundamental human capabilities that are central to body-self/body-world relations (Grigorovich & Kontos, 2018; Kontos et al., 2016). Finally, a human rights approach is particularly suited to considering sexual expression in the congregate care environment of RLTC as it recognizes the need to achieve a balance of rights and interests rather than automatically, and excessively, restricting any individual’s liberty for the purpose of protecting others (Grigorovich et al., 2019).

Drawing on a human rights approach to dementia and sexual expression, we explored the experiences of diverse professionals, family members, and persons living with dementia with explicit attention to the ways in which macro-level dynamics are influencing the support, or lack thereof, for sexual expression at the micro level.

### Policy context for the study

In Ontario, Canada, where this study took place, sexual expression of residents is regulated by a number of different types of laws and policies. For example, Ontario is subject to federal legislation (as well as case law) regarding sexual assault. This legal context requires that individuals demonstrate the capacity to communicate ongoing verbal consent to sexual activity. In instances where this mandated legal consent requirement cannot be met, the sexual act is treated as criminal (Goldberg, 2018; Grigorovich & Kontos, 2019). There is also provincial legislation that standardizes care and ensures accountability for residents living in RLTC and requires that homes operationalize codes of conduct through institution-specific policies and practices. While this legislation does not recognize explicitly that residents have sexual rights, it does recognize that they have the “right to exercise the rights of a citizen,” the “right to be afforded privacy,” as well as the right to “participate fully in making any decision concerning any aspect of his or her care” (Ontario Government, 2007, Par. II). Moreover, it requires that homes promote a zero-tolerance approach toward sexual abuse of residents and imposes a mandatory duty to report and investigate any unlawful conduct, harm or risk of harm, to residents, including sexual abuse. Without other types of policy that explicate the importance of also supporting sexual expression for health and well-being, compliance officers interpret the law to mean they are required to lay charges of neglect or abuse for sexual expression that may not be harmful or abusive. The definition of sexual abuse in the act is similar to how sexual assault is defined in the criminal code (e.g., “non-consensual touching and behavior or remarks of a sexual nature); no guidance is provided with respect to how to determine consent (nor does it help to identify what counts as sexual”). These critical variables are left up to individual homes/clinical experts to determine and to communicate to others, along with the requirement to have written a policy that details how reports of sexual abuse or neglect are investigated and addressed. Homes must also make their policies known to all staff, residents, and residents’ legally authorized decision makers. While this legislation requires that compliance inspectors conduct on-site inspections (which can be unannounced) to ensure that these requirements are met, little guidance is provided to homes on how to ensure that they are in compliance with respect to prevention of sexual abuse. The lack of guidance, together with the focus of inspectors on compliance with regulations, creates a climate of fear. The consequences of citation can include the temporary suspension of admissions to the RLTC home; RLTC homes thus tend to err on the side of overprotecting residents by restricting even positive sexual expression.

## Methodology

This was an exploratory qualitative research study guided by a critical interpretive approach (Denzin, 2017) and a human rights framework (Grigorovich & Kontos, 2018; Grigorovich et al., 2019; Kontos et al., 2016).

Participants were recruited using a combination of in-person (e.g., presentation about the study at local Alzheimer’s Society meetings) and online approaches (e.g., online professional listservs and dementia advocacy groups). All prospective participants contacted the first author directly either by email or telephone to learn more about the research project, to discover what participation would entail, and to have any questions answered. All participants provided written informed consent prior to participating in the research. In accordance with local ethical requirements, and with relational and process-based models of consent-seeking, participants living with dementia provided both written consent and their assent for each research encounter. Assent was determined based on interpretation of participants’ verbal and gestural cues as is deemed best practice when doing research with persons living with dementia (Dewing, 2007).

We recruited 27 participants. Of these, two were family members of persons living with dementia in long-term care (husband and daughter) and one was a family member to a person who used to live with dementia in long-term care before he passed away (wife). While we aimed to recruit persons living with dementia in long-term care and in community care, we were only able to recruit 3 persons living with dementia in the community (2 women and one man). We also recruited 21 diverse professionals involved in direct care and/or decision-making regarding intimacy and sexual expression in long-term care homes (i.e., nurses, physicians, therapists, social workers, bioethicists, and lawyers specializing in elder law).

### Data collection and analysis

Twelve professionals participated in a focus group, and interviews were conducted with a subgroup (*n*=7) in order to explore in greater depth key points of interest that were raised in the focus group discussions (e.g., capacity assessment). An additional 9 interviews were conducted with professionals who were unavailable to participate in a focus group. All family members and persons living with dementia separately participated in an interview. All focus groups were conducted in person, and interviews were conducted either in person or over the phone at the convenience of the participant. The same guide was used for the focus groups and interviews to explore perceptions of sexual expression (e.g., meaning and importance), identify factors regarding the support of sexual expression in RLTC, and examples of restricting or supporting intimacy or sexual expression in practice. In addition to the guide, a series of stock images of diverse examples of intimacy and sexual expression (e.g., image of an older man and woman kissing and image of two older women embracing) were used in the focus groups for precisely the same purpose.

All focus groups and interviews were digitally recorded and professionally transcribed verbatim. All of the data were analyzed using thematic analysis techniques (Denzin & Lincoln, 1998). Analysis began with independently reading the transcripts and identifying preliminary descriptive codes focusing on the identification of micro- (e.g., fear and forbidding hand-holding) and macro-level factors (e.g., professional mandates stigma), including examples of support or restriction of sexual expression. Drawing on a human rights approach, and analyses of public policies and professional standards with respect to sexual expression in RLTC in Ontario (Grigorovich & Kontos, 2018, 2019, 2020), the first and second authors then grouped codes into broader themes (i.e., “It’s natural but prohibited;” “Dementia and being old: The double stigma”; and “Discomfort, fear, and anxiety”) to capture the ways in which macro-level factors produce stigmatizing assumptions and attitudes and discriminatory responses to sexual expression at the micro-level of practice. These themes with illustrative quotes were then shared with the other authors for their review and comment, and then further revised.

## Findings

Our findings are organized thematically. “It’s natural but prohibited” captures the shared view of all participants that intimacy and sexual expression are important to the health and well-being of persons living with dementia and should be supported. It further captures the consensus of all stakeholders that sexual expression was very rarely supported in practice. This contradiction is taken up in subsequent themes that connect professionals’ attitudes and decision-making to macro-level factors, which produce discriminatory practices. Macro-level factors are captured with the theme “Dementia and being old: The double stigma,” which includes cultural stigma associated with dementia, and which is exacerbated by ageism, ableism, and erotophobia. These macro factors are reinforced by professional training that focuses on the prevention of inappropriate sexual behaviors, as well as legal and professional standards to safeguard residents from sexual abuse, whilst neglecting the importance of supporting positive sexual expression. Micro-level factors, which can largely be traced to stigma and other macro-level factors, were captured in the theme “Discomfort, fear, and anxiety” and included professionals’ negative attitudes toward sexual expression in the context of aging and dementia, their discomfort with facilitating intimacy and sexual expression in the context of their professional roles, as well as their anxieties regarding potential negative reactions from family members, or fear of professional or legal sanctions for failing to prevent abuse.

### It’s natural but prohibited

We found that there was consensus among all participants that intimacy and sexual expression are important to the health and well-being of persons living with dementia, and that this aspect of life should be supported in RLTC homes.

I think … as somebody ages, they don’t lose that need for sexual expression and intimacy … it’s natural, it’s part of being a human. (Professional, FG-2)

Interviewer: Is intimacy and sexuality an important part of your life?

Respondent: Oh, definitely. I think it’s a part of everybody’s life. At least intimacy. The sexuality comes for some people. But others, they can’t.

Interviewer: It’s true. Yeah. So, if you were living in a long-term care home, would you want nurses and other workers to have education in sexuality?

Respondent: I think it’s very important because some people have these notions that once you’re about 50 or past that, you’re going down in the grave – and you’re not. (Person living with dementia, I-24)

I worr[y] that she [doesn’t] get enough affection … I try to visit her twice a week but … if I’m the only one who’s hugging her where else does she get this, who else does she get this comfort [from] and I feel bad for her that she’s very lonely because she relies very much on touch now for communication. (Family member, I-12)

Despite the recognized importance of supporting intimacy and sexual expression for persons living with dementia, professionals noted that this was rarely explicitly supported in RLTC. The exception to this was the intimacy between heterosexual married couples.

And so, what you often find is that what people perceive as normal sexual activity is much less likely to be perceived as inappropriate … it’s not going to be interfered with. A very good example of that would be, say, a wife who lives in a long-term care home, who has Alzheimer’s, doesn’t recognize anybody at all. Husband comes in on Sunday afternoons, closes the door, and everyone knows they’re having sex in there. It’s like, well, [it’s] the husband, so I guess it’s okay … On the other hand, if it’s … outside of the norm, so it could be like anal sex, or gay sex, or something … those activities are much more likely to be perceived as inappropriate for older persons living in … long-term care. (Professional, I-07)

New relationships between residents living with dementia were explicitly prohibited whether these were heterosexual or not; this included restricting all forms of intimate physical contact between residents, ranging from hand holding, to kissing, and physical intercourse.

[W]e’re going to stop everything. I mean this is … common practice … I think there’s a big difference between penetrative intercourse and handholding [but] if they’re not married [to each other] then [we even] separate hands. (Professional, FG-2)

[I]n the majority of long-term care that I have worked in, [intimacy and sexual expression] is discouraged in any way, shape, or form. So, any kind of physical contact … if it was a heterosexual couple and they were married … [that’s] allowed … But if it was two females … or two males … [that’s] definitely not something that staff allow to happen. (Professional I-06)

Both persons living with dementia and family members agreed with professionals that intimacy and sexual expression were largely unsupported in long-term care. For example, when asked how moving into a RLTC home might affect their ability to be intimate or sexual with other people, a person living with dementia responded:

Respondent: They wouldn’t allow it

Interviewer: Can you tell me a bit more about that?

Respondent: Well, because I worked in dementia care I know … it is very much tabooed … and it should not be … Because they still have feelings and they … still need that connection. (Person living with dementia, I-26)

A family member reflecting on the RLTC home her mother is living in similarly noted how unsupportive it is of intimacy and sexuality:

[I]n her long-term care home everybody’s like all separated and disconnect[ed] … The physical environment design is like a hospital … if I’m in a hospital environment I don’t think of sexuality or intimacy … whereas if the environment was inviting, cheerful, that would create a different mood in the residents. (Family member, I-13)

### Dementia and being old: The double stigma

Persons living with dementia expressed the view that an ongoing challenge to experiencing intimacy and sexual expression was the stigma that is associated with dementia, which itself is part of the broader cultural stigma associated with disability, aging, and sexual expression. The following comments are particularly noteworthy in response to a question about pursuing romantic relationships:

Respondent: I have to be careful what I say, how I introduce myself, or things like that. I don’t want to scare them away.

Interviewer: Why do you think you would scare them away?

Respondent: Well, a lot of people don’t understand dementia, and they think that … you’re a loony, a crazy. They hear about stories of people being assaulted by a person living with dementia, that sort of thing.

Interviewer: Do they act differently when they find out?

Respondent: Oh yeah, they’re more standoffish, they sort of like keep their distance, and conversations are cut short. (Person with dementia, I-27)

I live alone. I’m a widow, so I’m on my own. But [intimacy and sexual expression] it’s still something that I think about, you know. I would like to have a companion, I would like to meet somebody. People around me discourage it. They think it’s very risky, that I could get taken advantage of and, you know, that it’s not – I’ve had someone say to me, “Well, it’s not fair to have a relationship with somebody with your diagnosis, because they start to care for you and your disease progresses and that’s not fair. You shouldn’t do that to anyone.” [But] I’m still a human being and I’m still very much alive … just because I have dementia doesn’t mean that I still don’t have that need and desire. (Person with dementia, I-26)

Stigma was also apparent in the attitudes held by many professionals that cognitive impairment erodes all capacity for decision-making and intentional self-expression. This prompted them to interpret all intimacy or sexual expression as “behaviors” and as pathological.

I can’t help but when I look at these pictures I think about how these different kinds of touch would be interpreted in the environment of long-term care, and I spend a lot of time reviewing charts and files of people that have been labelled as … exhibiting [sexual] behaviours … it just seems so natural and normal that people should interact in this way [but it] gets twisted or pathologized. (Professional, FG-1)

Yet, even a resident who would masturbate in the privacy of her own room was regarded as pathological. Because of her dementia and her advanced age, the frequency of her masturbation was thought to be abnormal and excessive:

[S]he wants to ... do it [masturbate] like on her own and in her room … it’s becoming often in the day … I don’t know how you would define a normal frequency in masturbation … Almost to a point where it would keep her up at night … even though she’s … 75 or 80 years old … I just don’t expect [someone] at that age will still do that ... our main concern was around like interfering with sleep sort of thing and then kind of worsening how she was during the day … to the point that it – like she doesn’t sleep at night at times, because she’s doing it and sometimes she doesn’t want to eat, because she’s doing it. So that’s the problem … we were looking [at] medications [for] dampening her sex drive … also our medical doctor ordered some medication, like vaginal, topical [cream] – maybe because she’s itching or whatever, but it didn’t work. So that means she’s not itchy down there, so that means something [else] going on, yeah … We worry about people being grumpy and irritable and then more uncooperative with the care that the nurses and staff need to provide … And that’s where we’re like covering all bases to see like is there anything that can make this person overall more comfortable and more cooperative. (Professional, FG-3)

Such views were reinforced by the education and training that they routinely received from psychogeriatric consultants to help them identify, document, and minimize internal biological (e.g., constipation and neuropathology) or external (e.g., noise and actions of professionals) “triggers” of “responsive behaviors” (BETSI Working Group, 2019). Within these curricula, sexual expression is identified as one type of “behavior” to be managed. This was reflected in how professionals characterized sexuality:

Interviewer: So, is sexuality just one more behaviour or do you think that there’s a qualitative difference between sexuality and other kinds of actions?

Respondent: I think it’s lumped in with behaviours because there’s kind of a trigger for it. There’s a need that’s not being addressed and therefore it comes up as behaviour. (Professional, FG-3)

So powerful is the stigma associated with dementia that professionals assumed that a diagnosis of dementia renders a person incapable of consenting to any intimate or sexual activity. Professionals thus interpreted all sexual expression as nonconsensual, necessitating restriction without conducting any assessment. Professionals, however, also acknowledged their lack of education and competence in this area, as well as a lack of access to other professionals who have the requisite education to do this.

Respondent: Well, my philosophy is that to whatever extent someone has capacity to make their own decisions, I think it’s our responsibility to support that.

Interviewer: Do you think that you have the skills and training to assess capacity in this regard?

Respondent: No. I don’t, and I don’t think many people feel comfortable assessing capacity in that regard. (Professional, I-12)

The stigma associated with dementia was exacerbated by ageism and erotophobia, which was reflected in professionals’ discomfort with their own parents’ sexuality, or the sexuality of older persons in general.

But then, you know, there’s also, never mind the stigma of dementia, there’s also just the simple stigma of being old. And I’ll confess that for most of my life the thought of my parents having sex … was something that I was never going to want to contemplate let alone have a conversation about with them. (Professional, I-07)

I think that there’s a stigma … I think because it’s a taboo topic it creates feelings of discomfort. (Professional, I-01)

### Discomfort, fear, and anxiety

There was the perception that sexuality was essentially a private matter and that being “exposed” to intimacy or sexual expression in the workplace compromised professionals’ personal beliefs.

I think it’s just sort of all the morality, you know, the morality and the sort of ethical sort of dilemmas that come up around sexuality. People have a lot of very strong opinions, sometimes rooted in religion or culture or upbringing, that really influence the way that we think about things like that. (Professional, I-06)

[W]e’re a multicultural facility ... Like different religions, different views on sexuality. And, for them, like seeing that is a taboo. “No, this is private. We can’t see this” … we have … nursing staff that’s been in that older generation and Orthodox Christian or Catholic and saying “This is disgusting. I don’t want this [resident]. Don’t assign this [resident] to me.” (Professional, FG-3)

Discomfort with the sexuality of residents was exacerbated when the sexual expression required some level of involvement by professionals; professionals viewed activities related to the support of intimacy and sexual expression as being distinct from other care activities that fall within their professional scope (e.g., cleaning of a pessary). This was reinforced by the absence of professional standards and competency expectation, education, and organizational policies that recognized or supported sexual rights. Facilitation of intimacy and sexual expression, as part of the work of care, therefore, was largely absent. The following discussion about a vibrator is illustrative of the prevailing challenges:

Interviewer: Do you think there’s greater discomfort around cleaning [a vibrator] versus cleaning anything else in that resident’s room?

Respondent 4: It’s not part of the nurse’s job. Whose job is it? Maintenance department? Housekeeping? It’s not in the [organizational] policies. Definitely it’s not the nurses’ job.

Interviewer: Do you think it’s different than some of the other kinds of interventions that sometime become part of your tasks?

Respondent 2: Right. You would clean up after a Fleet enema, you would clean up after, I don’t know … like pap smears. But like we had a client with a uterine prolapse that had a – what do you call it, what’s the thing they put in?

Respondent 1: Pessary.

Respondent 2: Pessary. I mean that didn’t have to come in and clean and all of that, but still there are other medical physical interventions that happen in the same area of the body, but why would this one be more?

Interviewer: Is it that this is not considered to be a “medical” intervention?

Respondent 3: A lot of people wouldn’t do that for themselves. A lot of people aren’t cleaning their own sex toys at home. They don’t have them. So now they’re coming to work and cleaning somebody else’s sex toys? It’s just not something we’re comfortable with. (Professional, FG-3)

Participants’ personal discomfort regarding explicit support of intimacy and sexual expression of residents was reinforced by their acute anxiety regarding negative reactions from family members if they did not restrict related activities or demonstrations of interest. A notable example of a negative reaction of a family member is the following where a professional describes being confronted by an irate daughter:

[There was] the woman who comes to visit her mother at the nursing home and is astonished to discover at 8:00 in the morning that there’s a man in her mother’s bed, and her astonishment and dismay is in part because her mother’s husband, her father, is alive and well and living in the community and here’s another man. This woman goes ballistic in the nursing home and starts screaming at people, “How can you let my mother lie in bed with that guy?” [and] saying her mother got raped. (Professional, I-02)

Interactions such as these were so negative that they left professionals reluctant to challenge family members even when they disagreed with their decisions to restrict intimacy or sexual expression, or believed these to be inconsistent with their professional judgment or the wishes and needs of the resident.

And they’re very firm and even though you express that they [two residents with dementia] have a relationship, they’ve bonded … they just don’t want it to continue and they say, nope our wishes are to separate them … Even just holding hands and the staff have to separate them. I have a hard time with that … I don’t agree that the family members are always [acting] in their best interest. (Professional, I- 16)

Yet, such negative interactions were also rarely recounted by professionals, and most family members themselves described a more positive attitude towards intimacy and sexual expression, including the formation of new relationships.

Interviewer: You mentioned that you think if this happened to your mom [in reference to her being found in bed with another resident] you would support it.

Respondent: For sure.

Interviewer: Is there anything that you would need to know if that happened?

Respondent: Just to let me know that that’s how they feel towards each other, so I don’t get surprised when I go like see them in one bed. Yeah, that I know that they like each other, and I just want to make sure that she’s not being taken advantage of or being manipulated in any way. As long as I know that she likes that, I would be fine. (Family member, I-13)

Even when family members were supportive, professionals were still reluctant to support this in practice. This suggests that professionals’ individually held perceptions, which themselves can be traced to broader cultural norms and assumptions regarding dementia and aging, were powerful barriers. They also reported receiving no education or guidance in this regard from their organizations or professional associations other than education on prevention of sexual abuse and the management of “inappropriate sexual behaviors.” This had the effect of reinforcing the belief that sexual expression was something from which the residents needed protection.

[T]here was a resident who was married living in the home and his spouse was not, and the resident developed a relationship with another female resident within the home that did develop into a sexual relationship; and this was reported … and the wife was okay with it ... she was completely fine with the relationship proceeding but ... the staff … wasn’t able to kind of get over that barrier. So, they were constantly trying to redirect the resident away from this female resident and interrupting their intimate moments together and reminding him that he had a wife. (Professional, I-020)

While professionals recognized that engaging family and residents more proactively in discussion about intimacy and sexual expression could help to prevent negative interactions with family members and better support residents, rarely was this opportunity presented.

Respondent: I think we would have a discussion with the families if we saw some signs that this was coming [intimacy between residents] or that this could potentially happen ... but I don’t think we address it – we wouldn’t address it until it got to the -- very close to actually happening.

Interviewer: Why do you think that you don’t address it until you think it’s closer to happening?

Respondent: Well I suppose it would be a big – it would be a lot of emotion and a lot of processing to happen for everybody, mainly for the staff and the family members. And then if it doesn’t actually happen then it’s kind of a lot of hoopla for nothing.

Interviewer: So, stuff like forming a relationship with another resident, is that something that you discuss with family members when they first bring their --?

Respondent: No, only if we see it happening.

Interviewer: Okay, so if it happens then you talk to them?

Respondent: Yeah, sort of reactive, not proactive. We’re not proactive …We’re trying to protect the family member…from the emotional hurt or the potential [of it]. (Professional, I-16)

Professionals’ anxieties regarding negative reactions from family members (if they did not restrict residents’ expressions of sexuality and intimacy) were tied to their fear that family members would perceive them as failing in their professional and legal obligations to protect residents from sexual abuse.

I think just generally not wanting to have those kinds of negative interactions with family members, they’re just not pleasant. And it makes you feel like you weren’t doing your job. Your job is to protect and keep people safe and so … you feel like you didn’t, you weren’t fulfilling your responsibility as an individual, as an organisation. (Professional, I-16)

There was also the fear of being reported by family members to the Ministry of Health and Long-Term Care, and the consequences of being held in breach of professional standards and legislation that emphasize that the primary obligation of professionals in the area of sexual expression is to safeguard residents from sexual abuse:

I think we’re all primarily concerned about whether we’re meeting our responsibilities in caring for our [residents] and maybe in terms of the legalities of it … What are the risks legally, for us as [an organization] and as an individual care professional? Because I mean there are high profile legal cases around sexuality and dementia … you assume it would never get to that, but there’s always the possibility. So, no one wants to be in that situation. (Professional, I-16)

[T]he sense I get when I talk to professionals [about supporting intimacy and sexual expression] is that it is this very acute anxiety around doing something that will put their licence at risk. (Professional, I-05)

## Discussion

Our analysis highlights an overwhelming consensus among participants that intimacy and sexual expression are aspects of life that are important to the well-being and quality of life of persons living with dementia and thus should be supported in RLTC homes. Research does link sexual expression in later life with multiple psychological and physiological benefits, including improved quality of life (Smith et al., 2019; Syme, 2014).Yet, our analysis also demonstrates that despite this consensus, intimacy and sexual expression were restricted rather than supported in practice. This was particularly the case in relation to intimacy and sexual expression outside of a heterosexual marriage. Wiskerke and Manthorpe’ review (2016) of research on new relationships arising between residents with dementia, whilst still married to another person, suggests that restriction of intimacy and sexual expression in this context may be connected to the perceived immorality of these relationships (specifically judgments about infidelity) by professionals, and the pressures exerted on professionals by family members who wish to be informed about such relationships. Further, other research suggests that professionals may perceive lesbian, gay, bisexual, trans and queer (LGBTQ) relationships and intimacies more negatively than heterosexual and cisgender ones and thus as more challenging to support in RLTC (Westwood, 2016). In our study, judgments about what was morally defensible were not the professionals’ main concern. Worries about any perception that they had failed to prevent sexual abuse created fear about family members’ anticipated reactions and the potential ramifications associated with their disapproval. In particular, professionals feared that family members would report them to the Ministry of Health and Long-Term Care for professional misconduct or that their facilities would incur sanctions if they supported intimacy and sexual expression of residents. It is curious that professionals did not express fear that families would report them to the police given legislative requirements regarding reporting of sexual assault (Ontario Government, 2007), especially as their fear of professional misconduct and sanctions was so great that they rarely engaged with families about this topic. On the other hand, families and persons living with dementia expressed the desire to be engaged and to be supported with respect to intimacy and sexual expression. When we consider the significant role of family members in decision-making regarding even the most intimate aspects of residents’ lives, and the importance of engaging persons living dementia about their own care (Read et al., 2020), this limited engagement is concerning.

Another significant micro-level factor expressed by professionals was their perception that cognitive impairment erodes all capacity for decision-making and intentional self-expression. With this assumption, all sexual expressions of persons living with dementia are interpreted as unintentional or non-consensual, effectively precluding any possibility of engaging in positive sexual expression. In our study people living with dementia expressed awareness of this prejudice about dementia and cited assumptions of incapacity as a key factor that restricts their pursuit of intimacy and sexual expression. These beliefs persist despite ample research that demonstrates that people living with dementia, even in the later stages of their illness, are capable of meaningful expression of choice and understanding. Further, with respect to most aspects of residents’ lives there is a legal and ethical obligation to determine incapacity before interfering with their expressed values and preferences (Brassolotto et al., 2020).

These micro-level factors offer only partial explanation of the restriction of intimacy and sexual expression; our analysis demonstrates that professionals’ perceptions and practices can largely be traced to the influence of macro-level factors. The most significant of these is the broader “cultural imaginary” associated with dementia (Gilleard & Higgs, 2013), which is reinforced by the “cerebralization” of selfhood and the biomedical discourse of dementia (Kontos et al., 2016). Together these discourses perpetuate a collective representation of dementia as a total erasure of self, which is part of a larger stigmatizing “decline narrative” of aging (Gullette, 2004). As a consequence, persons living with dementia are largely constructed as “unagentic” and “failed” aging subjects. In the context of sexuality, this stigma has produced the discourse of “inappropriate sexual behavior ” and the proliferation of professional and empirical literature focused on the management of sexual dysfunction of persons living with dementia and its prevention in RLTC (Grigorovich & Kontos, 2018; Kontos et al., 2016). This is reflected in “responsive behaviors” educational programs for dementia care professionals that has been widely implemented across Ontario (Grigorovich & Kontos, 2019; Gutmanis et al., 2015); “responsive behaviors” include any verbal or physical actions that are determined as being disruptive, intrusive, or harmful to persons living with dementia or others in their environment. Such actions are considered to be disease-driven “symptoms” that are a response to an internal biological (e.g., constipation and neuropathology) or external (e.g., noise and actions of professionals) “triggers” (DOS Working Group, 2019; Grigorovich & Kontos, 2019). The “responsive behaviors” philosophy was developed in an effort to challenge the biomedicalization of dementia care and the dominance of pharmacological interventions, by prompting professionals to shift from pathologizing the actions of persons living with dementia to understanding them as a meaningful response to a negative environment. Yet, despite this, the focus of education and practice remains at the immediate proximal level (e.g., at the micro-level of the actions of the individual resident or care provider) and on assessing, correcting, and controlling “behaviors” of individuals rather than altering the broader social and physical environment. For example, within “responsive behaviors” educational curriculums, sexual expression is identified as a behavior that may be risky (e.g., “sexual expression of risk”) if it is “intrusive or without the knowledge/consent of the individuals involved” (DOS Working Group, 2019). While professionals are instructed to assess whether the sexual expression should be accommodated or restricted based on the risk it poses to self and others, given that professionals rarely receive any education on the benefits of sexual expression or how to support it (Grigorovich & Kontos, 2019), “responsive behaviors” training prompts them to focus exclusively on management and restriction with the goal of protecting residents and others.

Another key macro-level factor that influences professionals’ micro-level decision-making and attitudes, are discriminatory and paternalistic laws and policies that prioritize prevention of sexual abuse while entirely neglecting the importance of supporting positive sexual expression (Ontario Government, 2007). In restricting sexual expression based on cognitive capacity to consent and emphasizing protection over self-determination, such laws and policies reinforce ageist, ableist, erotophobic, and oppressive sexual norms and legitimize material forms of discrimination, including increased professional and familial surveillance of sexual expression and its behavioral and pharmaceutical restraint (Grigorovich & Kontos, 2020; Sandberg et al., 2020). In the context of our study, professionals certainly acknowledged that they should establish whether or not a sexual expression is nonconsensual prior to interfering with this, yet they rarely did so. They not only lacked the requisite training but also had no access to professional resources that could facilitate the assessment of this type of capacity. All of these macro factors offer explanations for the seeming tension between professionals’ understanding of sexual expression as important to support, yet also as something that they should restrict for persons living with dementia in RLTC. The exclusive focus on protection and restriction denies persons living with dementia the “dignity of risk” by holding them to a higher standard of decision-making than persons without cognitive disabilities, who are generally allowed to make sexual decisions without first “weighing the pros and cons or … implications of their decisions” (Lindsay, 2010, p. 314). In the absence of education and policies for professionals that recognize the importance of supporting intimacy and sexual expression for residents of RLTC homes, and clear guidance in how to do this, professionals (and organizations) will continue to perceive these issues as complex and fraught with peril, and thus best avoided (Gilmer et al., 2010).

While some research suggests that more professional education will lead to greater support of residents’ sexual expression in practice (Aguilar, 2017; Mahieu et al., 2016), our analysis complicates what appears to be a linear relationship between knowledge and practice. While professionals understood that supporting sexual expression was important, this was at odds with their personal values (e.g., sexuality is a private matter), and also with a broader cultural stigma regarding dementia and the care of older adults. Thus, understanding was not enough to change restrictive practices. An important contribution of our analysis is its demonstration of the interrelationship between professionals’ education on management of “responsive behaviours” and current policies and legal standards which reproduce and reinforce the cultural stigma associated with dementia and aging, and undermine support for positive sexual expression as part of the duty of care in RLTC. Given this complexity, education must address personal beliefs and cultural stigma. Yet, where negative perceptions and prejudice are mentioned in existing education and training, professionals are merely instructed to confront these, which leaves unexplored how individual and interpersonal-level stigma is generated and reinforced by cultural stigma and other macro-level factors (e.g., professional standards, organizational policies, and philosophy of care) (Bamford, 2011). Moreover, approaches to education and training have predominately entailed passive forms of dissemination (e.g., publication) despite research that indicates that knowledge translation is more successful when it consists of multifaceted, multi-component, and interactive educational interventions (Kontos et al., 2020; Kontos & Poland, 2009). Specifically, there is evidence to suggest that interactive arts-informed educational interventions can result in significant professional practice and policy changes in RLTC homes (Basting et al., 2016; Kontos et al., 2020).

To enable culture change in RLTC, professional education and practice development initiatives must be complimented by the creation of public policies that recognize the positive obligation to support access to uncoerced and pleasurable sexual expression, and that do not undermine the formation of intimate and sexual relationships. Such changes would ensure that persons living with dementia in RLTC are guaranteed the social and environmental conditions necessary for them to realize their sexual rights and would also reduce professionals’ fears that supporting sexual expression will be perceived as professional misconduct. As we have argued elsewhere, the creation of new policies must be grounded in the recognition that sexuality is fundamental to embodied self-expression and relationality, which are primary means of engagement for persons living with severe and persisting cognitive impairment such as dementia (Grigorovich & Kontos, 2018; Kontos et al., 2016). This could entail challenging or amending existing laws and policies to ensure that they are not overly restrictive of voluntary and uncoerced sexual expression, and that they are consistent with human rights law around disability that enables access to mechanisms of supported decision-making so that persons living with dementia can exercise and enjoy the right to legal capacity (De Sabbata, 2020; UN General Assembly, 2007). One approach to this could include the creation of legal documents that enable people living with dementia to communicate their values, wishes, and preferences regarding sexual expression, and to select another person to make decisions on their behalf should they become incapable. Such documents would of course need to be implemented alongside the creation of appropriate safeguards to ensure that they do not contribute to injury or other harm to the person or others (Boni-Saenz, 2016; Sorinmade et al., 2020). While we acknowledge that the creation and operationalization of such documents and safeguards will be ethically, legally, and professionally challenging, it is consistent with Ontario’s approach to supporting other types of decisions of legally incapable persons (e.g., in regard to medical treatment) and we believe that it is an important step toward recognizing the sexual rights of persons living with dementia. Finally, our analysis further suggests the need for the creation of new policies and laws for RLTC based on an affirmative duty to create access and opportunity for persons living with dementia in these spaces to make decisions around sex, intimacy, and whether to engage in intimate and sexual relationships. Such duties would require that RLTC homes address implicit and explicit prohibitions including the design of the built environment, that unfairly restrict wanted sexual expression (e.g., the practice of assuming rather than assessing incapacity for consent, imposing unreasonable restrictions on residents’ purchase and use of sexual materials, and the establishment of barriers to sexual privacy such as failing to knock on doors before entering resident’s rooms and failing to provide private spaces for sexual expression). Instituting such duties would additionally require that providers and RLTC homes actively facilitate sexual expression and incorporate this into care planning through the provision of sexual education and counseling for residents and family members and the creation of opportunities for the formation of sexual and intimate relationships. Examples of the latter could range from developing and hosting diverse social events and romantic outings for residents, to enabling residents to engage the services of sexual surrogates or other types of professionals that provide sexual services (Grigorovich & Kontos, 2018; Henrickson et al., 2021; Kontos et al., 2016). These examples are drawn from international RLTC homes and have yet to be implemented in Ontario, or anywhere else in Canada to our knowledge.

### Limitations and future directions

Although our intention was to include an equal number of participants who live with dementia and family members, we ended up with only a select few from these two important stakeholder groups. This was largely a function of our reliance on a partner organization to assist with recruitment. Unfortunately, that organization experienced significant and unexpected restructuring at the time of our study. The few participants we were able to recruit from these stakeholder groups noted their strong desire for support of sexual expression in RLTC. Given that this finding is inconsistent with some research in this area (Bauer et al., 2013), it will be important to engage more individuals from these groups, and in particular persons living with dementia in RLTC in order to explore whether our findings were anomalous or if others share this perspective. Moreover, while our analysis suggests that LGBTQ intimacies and relationships are perceived particularly negatively by professionals, findings which are consistent with other research in this area (Wiskerke & Manthorpe, 2016), we did not recruit specifically for members of these communities, nor did we explicitly explore with participants their perceptions about their own sexual identity or expression, or that of others. Given that participants’ reflections suggest that they may conflate sexual expression and sexual identity, particularly in relation to considering the needs of LGBTQ people living with dementia, this will be another important consideration in future research.

## Conclusion

Our findings demonstrate the importance of understanding and redressing macro-level barriers that contribute to the stigmatization of persons living with dementia in RLTC and the restriction of their sexual rights. In particular, we have identified the importance of challenging the cultural stigma associated with dementia, disability, and aging, as well as paternalistic policies and practices that presume that persons living with dementia lack the capacity for sexual decision-making and the formation of intimate relationships. We have called attention to a complexity that has enormous potential to inform the development of educational initiatives and suggests a need for legislative and organizational policy changes to ensure that persons living with dementia have equal opportunities to pursue positive sexual expression to the greatest extent possible.
